# A Natural Gas Fermentation Bacterial Meal (FeedKind^®^) as a Functional Alternative Ingredient for Fishmeal in Diet of Largemouth Bass, *Micropterus salmoides*

**DOI:** 10.3390/antiox11081479

**Published:** 2022-07-28

**Authors:** Boyuan Guo, Xia He, Chunyu Ge, Min Xue, Jia Wang, Matt Longshaw, Jie Wang, Xiaofang Liang

**Affiliations:** 1 National Aquafeed Safety Assessment Center, Institute of Feed Research, Chinese Academy of Agricultural Sciences, Beijing 100081, China; guoboyuan2023@163.com (B.G.); hehehexia43942@163.com (X.H.); gechunyu@caas.cn (C.G.); xuemin@caas.cn (M.X.); 2 Calysta (China) Company Limited, Shanghai 200041, China; wangjia@calysta.com; 3 Calysta (UK) Company Limited, Redcar TS10 4RF, Cleveland, UK; mlongshaw@calysta.com; 4 Feed Processing and Quality Control Innovation Team, Institute of Feed Research, Chinese Academy of Agricultural Sciences, Beijing 100081, China

**Keywords:** largemouth bass, methanotroph (*Methylococcus capsulatus*, Bath) bacteria meal (FeedKind^®^), growth performance, antioxidant capacity, intestinal health

## Abstract

A 10-week growth study was conducted to evaluate the effect of a natural gas fermentation bacterial meal (FeedKind^®^, FK) as a fishmeal (FM) alternative in largemouth bass (*Micropterus salmoides*) (48.0 ± 0.03 g). Four isonitrogenous and isoenergetic diets were formulated including one commercial control (C, 42% FM) and three experimental diets with gradient FK of 3% (FK3, 29%FM), 6% (FK6, 26%FM) and 9% (FK9, 23%FM), respectively. FK-fed groups showed significantly higher SR than that of C group. The WGR and SGR of fish fed FK3 and FK6 were significantly higher than those of FK9, but not statistical different from the C group. FK-fed groups showed higher apparent digestibility coefficients of dry matter and nutrients. Further, FK-fed groups increased the ratio of SOD/MDA in the plasma and liver, and the upregulation of intestinal *Keap1* and downregulation of HIF1α was found in FK3. Furthermore, FK-fed groups showed higher microbial richness and diversity. Pearson correlation analysis found that antioxidant relevant biomarkers were negatively correlated with the relative abundance of certain potential beneficial bacteria. In conclusion, supplemented up to 3–6% FK replacing FM in a low FM diet of largemouth bass could increase growth, survival rate, antioxidant capacity, and improve gut microbiota.

## 1. Introduction

The expansion of aquaculture has led to the increase in demand for fishmeal (FM), due to its high protein content, balanced amino acid profiles, high digestibility and good palatability [1,2]. It is recognized that the cost of FM derived from wild-captured forage fish is variable, and, although sustainable at current usage levels, there is limited scope to increase the amount of FM from wild caught sources to satisfy the requirement of the increasing development of aquaculture [3]. The gradually increased cost of FM leads researchers and feed manufacturers to find alternative proteins for sustainable aquaculture [4,5]. Until now, some alternatives from oil plants or terrestrial animal by products, such as meals or protein concentrated from soybean, rapeseed, cottonseed and cereals, meat by-products etc., have been widely tested as protein sources in aquafeed [6]. More recently, some novel protein sources, such as insect meal [7] and single-cell protein (SCP), have been developed and tested in light of higher environmental consciousness and the use of feedstocks from renewable or recycled sources [8].

Under the dual demand for food security and carbon neutrality, the SCP obtained from bacteria, fungal and microalgae by recycling waste materials (e.g., straw fibers, molasses and waste green-house gas) have the great potential to supply the protein sources with high digestibility, balanced nutrient profile and along with some functional components, such as nucleotide and water-soluble metabolites [9]. Methane, as a greenhouse gas source, is the major component of natural gas and has been considered an attractive substrate for single cell protein production [10,11]. As one of the commercial bacterial protein meals, FeedKind^®^ (FK) (Calysta, Inc., San Mateo, CA, USA) is produced by the continuous gas fermentation using methane as a single carbon source, and ammonia as a nitrogen source by *Methylococcus capsulatus* Bath, *Cupriavidus* sp., *Aneurinibacillus danicus*, and *Brevibacillus agri.* [12]. It is a reddish/brownish meal with approximate 70% crude protein, 10% lipids, 7% ash and well-balanced amino acid profile [13,14,15]. It was previously developed under the trade name Bioprotein ^®^ and initially tested in fish feed by Skrede et al. in 1998 [14]. To date, there have been more than 20 publications that demonstrate that these methane-oxidizing bacteria meal are safe, effective and with low carbon footprint in livestock and aquatic animal feed [9,16]. In general, FK can be used at 4~13% of total diet to replace equivalent amount of FM in fish and shrimp feed with positive or equivalent effects on survival and growth performance [17,18,19,20,21,22]. Interestingly, the inclusion of 300 mg of *M. capsulatus* per kg of feed could prevent the development of dietary soybean meal induced intestinal inflammation in Atlantic salmon [23,24,25].

Largemouth bass (*Micropterus salmoides*) is an important freshwater carnivorous fish species native to lakes and small rivers in North America [26], the trophic level is 3.8 from FISHBASE database (https://www.fishbase.se, accessed on 20 July 2022). Nowadays, it has become an important economic farmed freshwater species in China [27]. Chinese aquaculture production of largemouth bass reached 600 thousand tons in 2021 [28]. Largemouth bass is a representative species that successfully changed to be fed artificial diet from the trash fish during the decade in China. As a typical carnivorous fish, largemouth bass showed high requirements for feed protein and energy in the commercial diet, which usually contained 46–51% crude protein and 12% crude fat based on optimal nutritional requirement studies [29,30]. In our previous studies, we have established a serial of recipe with several alternative proteins, such as cottonseed protein concentrated (CPC), *Clostridium autoethanogenum* protein (CAP) and soy protein concentrated (SPC) with minimized 28–30% FM [31,32,33]. However, quite a few feed manufactures are using high FM (40%) still in the commercial recipes for this species.

Collectively, the objectives of this study were to investigate the effect and possibility of various levels of FK to further reduce the FM content in the diet of largemouth bass by evaluating the growth performance, antioxidant, immunity responses, and microbiota in distal intestine of largemouth bass.

## 2. Materials and Methods

### 2.1. Growth Trial and Sample Collection

FeedKind^®^ (FK) was supplied by Calysta, Inc (San Mateo, CA, USA) and consisted of inactivated and dried biomass of the aerobic bacteria *Methylococcus capsulatus* (Bath), *Cupriavidus* sp., *Aneurinibacillus danicus*, and *Brevibacillus agri*, utilizing natural gas as the carbon and energy source. *Methylococcus capsulatus* represents around 92–94% of the bacterial biomass with the other bacteria making up the rest of the product. Steam dried FM (Prime level) and fish oil was supplied by Tecnológica de Alimentos S.A., Ltd. (San Borja, Lima, Peru). Soy protein concentrated (SPC), soybean meal and soy oil were supplied by Bohi Industry Co., Ltd., Shandong, China. Other ingredients were bought from a local market.

Four isonitrogenous and isoenergetic diets were formulated including one control (C, 42% FM and 15% SPC), and 3 low FM diets with 25% of SPC and gradient FK of 3%, 6% and 9% to further replace FM, respectively (referred as FK3, FK6 and FK9), with balanced essential amino acids, fatty acids and available phosphorus. Each diet was processed into 3 mm diameter floating pellets under extrusion condition as previous description [34]. All diets were air-dried at room temperature (26–30 °C) and stored at −20 °C until required. The diet formulation and analyzed chemical compositions are shown in Table 1. The analyzed amino acid composition of experimental diets is shown in Table 2.

### 2.2. Experimental Fish, Feeding and Sampling

Juvenile largemouth bass were obtained from a single-size-graded population (Tianjin Yuqing Aquatic Technology Company, Tianjin, China). The experiment was conducted in the indoor circulating water system at the National Aquafeed Safety Assessment Center of IFR, CAAS (Beijing, China). All fish were acclimated and fed the commercial diet (51% crude protein, 12% crude lipid) for 2 weeks before the trial. Fish with initial body weight of 48.0 ± 0.03 g, were selected and distributed into 16 tanks (256 L) with 25 fish/tank and four replicates each treatment. Largemouth bass were fed twice daily at 08:00 and 17:00 to apparent satiation for 70 days. The water temperature was maintained at 25–27 °C, pH = 7.5–8.5, dissolved oxygen (DO) > 7.0 mg/L and NH4-N < 0.3 mg/L. The photoperiod was 12L: 12D and the light intensity was 400 lx.

At the end of the trial, fish of each tank were batch weighted. The chyme from the distal intestine of 6 individual fish/dietary treatment was randomly collected at 3 h postprandial, and then snap-frozen in liquid nitrogen before stored at −80 °C for microbiota analysis. In this case, 12 fish of each treatment (3 fish from each tank) were randomly selected and anesthetized with chlorobutanol (300 mg/mL) for sampling at postprandial 24 h. The body weight, body length, viscera weight, liver weight, and abdominal adipose weight were recorded individually to calculate fish condition factor (CF), viscera somatic index (VSI), visceral adipose index (VAI), and hepatosomatic index (HSI). Blood was collected from the caudal vein and centrifuged at 4000× *g* for 10 min at 4 °C to obtain plasma and then stored at −80 °C for analysis of hematological parameters. The distal intestine tissues were collected and separated as two parts for histology examination (fixed in 4% paraformaldehyde solution), and RNA isolation, respectively. All samples were stored at −80 °C until analysis. Three fish per tank were collected for the assay of whole-body compositions. The liver tissue of each tank was pooled and freeze-dried for hepatic lipid content determination.

Nutrient apparent digestibility of the diets was assessed using the indirect method using 0.1% Y_2_O_3_ as an inert marker. Feces collections were initiated 1-week after the formal feeding trail, which allowed sufficient time for all the gut content from any previous diet to be purged as described by Wang et.al. (2012) [35]. Feces were collected using a settling column that separated the fecal material from the effluent water and then stored at −20 °C for subsequent analysis. To minimize nutrient leaching in the feces, only intact feces that was produced 1 h after meals were collected. Daily fecal samples from each tank were pooled over the course of the experiment until a sufficient sample was available for chemical analyses. During the feeding period, the daily feed consumption was recorded by removing the unconsumed feed 1 h later after feeding and dried to constant weight at 70 °C and weighed. Leaching loss of the unconsumed diet was estimated by leaving 20 pellets of each diet in tanks without fish for 1 h, then recovering, drying and reweighing.

### 2.3. Chemical Analysis

The macro nutrient composition analyses of the diets and feces were carried out in duplicate according to AOAC, 2019 [36]. The dry matter, crude protein, crude lipid, ash and gross energy were conducted according to standard methods as previously described [37]. The copper content of diets was analyzed in triplicate using graphite furnace atomic absorption spectrum. The inert marker (Y_2_O_3_) in diets and feces was analyzed using inductively coupled plasma atomic emission spectrometry. The amino acid contents of diets and feces were analyzed at Amino Lab, Evonik Industries (Beijing).

### 2.4. Liver Function, Metabolites and Immunity Response in Plasma and Distal Intestine Tissue

The plasma alanine aminotransferase (ALT), aspartate aminotransferase (AST), total bile acid (TBA), alkaline phosphatase (AKP), glucose, total protein content (TP), Triglyceride (TG), total cholesterol (TC), high-density lipoprotein cholesterol (HDL-C), low-density lipoprotein cholesterol (LDL-C), malondialdehyde (MDA), and superoxide dismutase (SOD) were determined by assay kits (Nanjing Jiancheng Co., Nanjing, China) following the protocols given by the supplier. The content of plasma non-esterified fatty acid (NEFA) was determined by the assay kit (Wako Pure Chemical Industries, Ltd. Tokyo, Japan). The plasma reactive oxygen species (ROS), lipopolysaccharide (LPS), complement C3 (C3), immunoglobulin M (IgM), and distal intestinal interleukin 1β (IL1β), tumor necrosis factor α (TNFα), interleukin 10 (IL10) were detected by enzyme-linked immunosorbent assay (Jiangsu Meimian industrial Co., Ltd.; Yancheng, China).

### 2.5. Histopathological Examination of the Distal Intestine

The fixed distal intestine samples were dehydrated by the standard procedures, and the samples were embedded in paraffin and cut to 6μm sections. The distal intestine was stained following the protocols of hematoxylin and eosin staining (H&E) and Alcian blue staining, Images of the stained sections were acquired with TissueFAXS Histo S, TissueGnostics GmbH, Vienna, Austria).

### 2.6. Quantitative Real-Time PCR

Total RNA isolation, quantification, reverse transcription and mRNA quantification of distal intestine were conducted following the protocols as previously described [34]. EF1α (elongation factor 1α), whose expression was unaffected by the treatment in the present experiment, was used as an endogenous reference to normalize the template amount. The gene-specific primers used for mRNA quantification by RT-qPCR are shown in Table 3.

### 2.7. Western Blot

Distal intestine was homogenized in RIPA buffer (Beyotime, Shanghai, China) with an added phosphatase inhibitor cocktail (CST, Boston, MA, USA). The protein concentration was measured using a BCA Protein Quantification Kit (Bio-Rad, Hercules, CA, USA). The primary antibodies against HIF1α (BS3514, Bioworld, Nanjing, China) and β-tubulin (loading control, #2146, CST, Boston, MA, USA) were infused to probe target proteins prior to protein visualization using a horseradish peroxidase secondary antibody (ProteinSimple, San Francisco, CA, USA). Automated Western blots were performed on a Jess ^TM^ system (Protein Simple, San Francisco, CA, USA) using pre-filled plates (12–230 kDa) according to the manufacturer’s standard instructions.

### 2.8. Gut Microbiota

About 100 mg distal intestinal chyme from each fish were used for DNA extraction based on the manufacturer’s instructions from the E.Z.N.A. ^®^ soil DNA Kit (Omega Bio-tek, Norcross, GA, USA). Purity and quality of the genomic DNA were checked on 1% agarose gels. The PCR amplification of bacterial 16S rRNA gene (V3-V4 hypervariable region) were amplified using the universal primers 336F (5′-GTACTCCTACGGGAGGCAGCA-3′) and 806R (5′-GTGGACTACHVGGGTWTCTAAT-3′). The PCR amplification of 16S rRNA gene were run in triplicate. After the PCR amplification, the quality of PCR products was evaluated for library preparation on 1.5% agarose gels and the samples with a bright band of size between 300 bp and 350 bp were used for further analysis. Before library preparation, the PCR products were purified according to manufacturer’s instructions of AxyPrep DNA Gel Extraction Kit (Axygen Biosciences, Union City, CA, USA), and then quantified using Quantus ™ Fluorometer (Promega, Madison, WA, USA). The Illumina MiSeq PE300 platform (Illumina, San Diego, CA, USA) was used for the library preparation and sequencing based on the standard protocols.

The raw 16S rRNA gene sequencing reads were demultiplexed, quality-filtered by fast version 0.20.0, and merged by FLASH version 1.2.7 [38]. Operational taxonomic units (OTUs) with 97% similarity cutoff [39,40] were clustered using UPARSE version 7.1 [40], and chimeric sequences were identified and removed. The taxonomy of each OTU representative sequence was analyzed by RDP Classifier version 2.2 [41] against the 16S rRNA database (e.g., Silva v138) using a confidence threshold of 0.7. Differences between dietary treatments in alpha diversity were evaluated by four indices: (1) Observed and Chao 1 indices, which counts the numbers of ASV in each sample indicating microbial richness; (2) Shannon and Simpson indices which takes into account richness and how many of each ASV are observed, indicating microbial diversity. Notably, the high Shannon indices value means high microbial diversity, while the high Simpson indices value indicates low microbial diversity. Regarding of beta diversity of microbiota, two indices were used to estimate the phylogenetic difference between treatments: (1) Unweighted UniFrac Distance, which takes into account the number of different ASV and their phylogenetic distance; (2) Weighted UniFrac Distance, indicating the number of different and similar ASV, as well as their phylogenetic distance.

### 2.9. Statistical Analysis

All data were reported as the mean value with the standard errors of the mean (mean ± SEM). The data except microbiota data were evaluated for normal distribution and homogeneity of variance using Shapiro-Wilk test and the Barlett’s, respectively. A one-way analysis of variance (ANOVA), followed by Duncan’s multiple comparisons was conducted by SPSS 25.0 (IBM SPSS Statistics, Armonk, NY, USA). Differences were regarded as significant when *p* < 0.05. Permutation multivariate analysis of variance (PERMANOVA) was performed with 999 permutations to the unweighted and weighted UniFrac distance matrix results from the beta diversity analysis of QIIME 2. Group differences in relative abundance of microbiota at the phylum and genus taxonomic level were tested using the nonparametric Kruskal-Wallis test followed by Dunn’s multiple comparisons. All the graphics were drawn by GraphPad Prism 8.0 (GraphPad Software Inc., San Diego, CA, USA). The Pearson correlation between the gut microbiota and macro biomarkers from the same tank was analyzed using GraphPad Prism 8.0. Values marked with symbol “*” are significant correlations (*p* < 0.05), and “**” are very significant correlations (*p* < 0.01).

## 3. Results

### 3.1. Growth Performance and Morphometric Parameters

**Figure 1:**
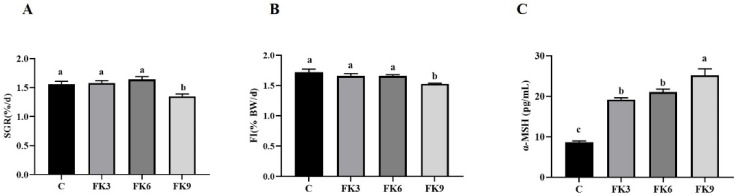
Specific growth rate ((**A**), *n* = 4), Feed intake ((**B**), *n* = 4), and plasma anorexia hormone α-MSH content ((**C**), *n* = 8) of largemouth bass fed diets with various FK levels (mean ± SEM). SGR (specific growth rate, %/d) = 100 × [ln (final body weight) − ln (IBW)]/d]; FI (feed intake, %) = 100 × feed intake/[(W_f_ + W_i_)/2]/days; W_f_: final total weight, W_i_: initial total weight. Superscript lowercase letters “a, b, c” indicate significant differences (*p* < 0.05) among groups.

The results of growth performance and morphometric parameters are presented in Table 4 and Figure 1. All FK inclusion groups showed significantly higher survival rate (SR, 94%~96%) than that of the C group (85%, *p* < 0.05). The weight gain rate (WGR) and specific growth rate (SGR, Figure 1A) of fish fed diets FK3 and FK6 were significantly higher than that of FK9 group, but not significantly different from the C group. The FK9 group showed the lowest growth performance, which might be related to the lower feed intake (FI) of this group (Figure 1B, *p* < 0.05). The α-MSH, hormones that suppress feed intake in the central nervous system, gradually increased with replacement levels (*p* < 0.05) (Figure 1C). The feed conversion ratio (FCR) ranged from 1.13–1.22, and there were no significant differences among the four groups. There were no significant differences in the productive protein value (PPV). Productive lipid value (PLV) in the FK3 and FK6 groups were significantly higher than in the FK9 group (*p* < 0.05), but no significant difference from the C group (Table 4).

### 3.2. Macro-Nutrient Compositions and Copper (Cu) Content in Whole Body, Liver Tissue and Feces

There were no significant differences in whole body moisture, crude protein, and crude lipid content among treatment groups (*p* > 0.05, in Table 5). The ash content of whole fish increased clearly with dietary the increasing level of FK. With dietary increasing level of Cu from 3.88 mg/kg in the control group to 16.4 mg/kg in the FK9 group (Table 1), we observed that Cu was not accumulated in whole body or in liver, but largely discharged with feces (*p* < 0.05, in Table 5).

### 3.3. The Apparent Digestibility Coefficients (ADC) of Dry Matter, Protein, Energy and Cu in Test Diets

In general, FK inclusion diets showed higher ADC of dry matter, and FK3 and FK6 groups showed significantly higher ADC of protein (ADC_p_) and energy (ADC_e_). Furthermore, the FK groups showed much higher Cu digestibility than that of the C group (*p* < 0.05, in Table 6).

### 3.4. Liver Function and Metabolites in Plasma

The liver function and metabolites in plasma of largemouth bass are presented in Table 7. Improved liver function, as denoted by the significant decrease in plasma ALT, was observed in the FK3 and FK6 compared to the higher level of plasma ALT in the C group and the intermediate level in the FK9. The FK6 group also had a significantly lower plasma TBA (*p* < 0.05). There were no differences in plasma AST and AKP among groups.

Higher plasma glucose and lower TP in the FK6 group was observed (*p* < 0.05). The metabolites of lipid metabolism, including metabolites of fatty acids, triglyceride and cholesterol were generally at homeostasis status among groups at postprandial 24 h.

### 3.5. Histopathology and Systematical Antioxidant Responses

**Figure 2:**
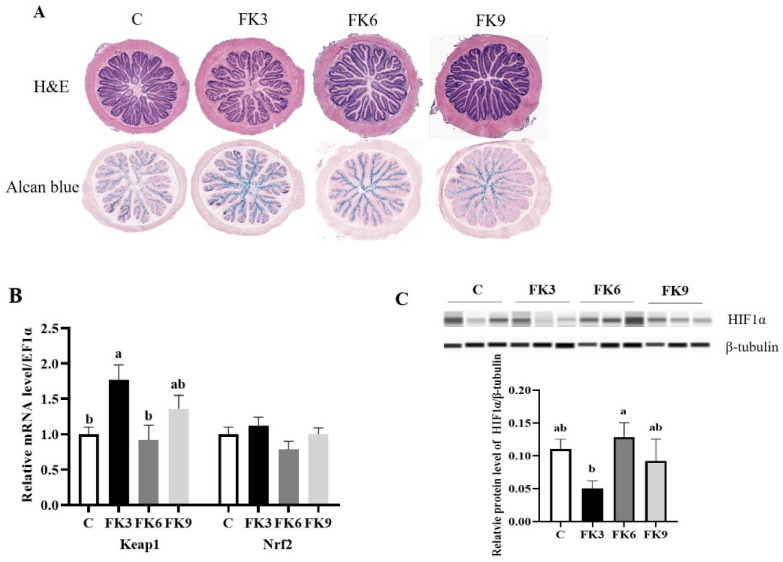
The effects of dietary various FK levels replacement on histology and antioxidant responses of distal intestine of largemouth bass. (**A**) The phenotypes of distal intestinal histopathological examination by H&E and Alcian blue staining (*n* = 12); (**B**) The mRNA levels of *Keap1* and *Nrf2* (*n* = 12). (**C**) The relative protein level of HIF1a (*n* = 3). Different superscript letters “a” or “b” indicate significant differences (*p* < 0.05), whereas the same superscript letters show no significant differences among groups (means ± SEM).

The sections of distal intestine were examined after H&E and Alcian blue staining. In this case, 12 samples were observed in each group, and no obvious abnormalities were observed in the distal intestine of largemouth bass (Figure 2A). However, the antioxidant gene *Keap1* was upregulated at mRNA level and the HIF1α protein expression in distal intestine tissue was downregulated in the FK3 group (Figure 2B,C, *p* < 0.05). Additionally, the plasma and hepatic MDA were significantly reduced, and hepatic SOD increased in FK groups. Both in plasma and liver tissue, SOD/MDA ratio were significantly increased in FK groups compared to that of the fish fed control diet (*p* < 0.05 in Table 8).

### 3.6. Immunity and Inflammatory Responses

**Figure 3:**
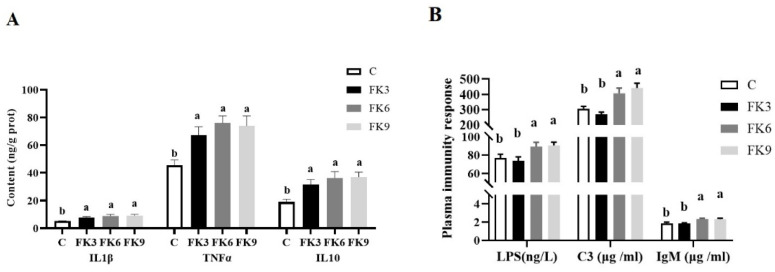
Inflammatory and immunity response of largemouth bass fed diets with various FK levels. (**A**) The intestine mucosal inflammatory cytokines contents; (**B**) Plasma LPS, C3 and IgM contents. Different superscript letters “a” or “b” indicate significant differences (*p* < 0.05), whereas the same superscript letters show no significant differences among groups (means ± SEM, *n* = 12).

With the exception of LPS and complement C3 in the FK3 group, pro- and anti-inflammatory and immune responses were elevated in the FK groups. In particular, the proinflammatory cytokines IL1β, TNFα, and the anti-inflammatory cytokine factors IL10 in the distal intestinal of the FK groups were significantly increased compared to the C group (Figure 3A, *p* < 0.05). The immunity parameters, including plasma LPS, complement C3 and IgM of FK6 and FK9 groups were significantly higher than those in the control and FK3 group (Figure 3B, *p* < 0.05).

### 3.7. Energy Metabolism in Distal Intestine

**Figure 4:**
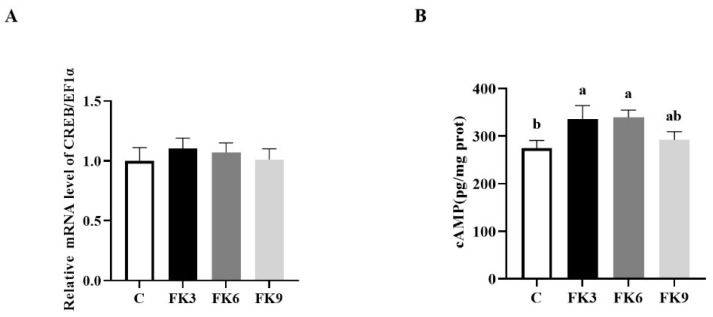
The energy metabolism in distal intestine of largemouth bass fed diets with various FK levels. (**A**) CREB; (**B**) cAMP. Different superscript letters “a” or “b” indicate significant differences (*p* < 0.05), whereas the same superscript letters show no significant differences among groups (means ± SEM, *n* = 12).

The CREB and cAMP were energy metabolism biomarkers. The *CREB* mRNA level was not significantly different among groups (Figure 4A), but the second massager cAMP content in distal intestine was significantly increased in the FK3 and FK6 groups (Figure 4B, *p* < 0.05).

### 3.8. Gut Microbiota

**Figure 5:**
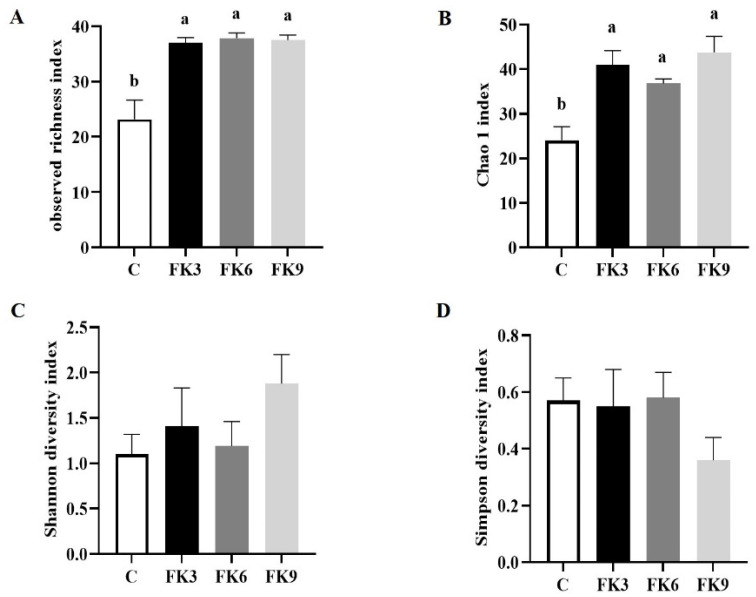
The alpha diversity of microbiota in the distal intestinal of largemouth bass fed diets with various FK levels. (**A**) Observed richness; (**B**) Chao index; (**C**) Shannon index; (**D**) Simpson index. Graph bars labelled with different letters “a” or “b” on top indicate significant results, whereas bars with the same letter correspond to results that show no significant differences among groups (*p* < 0.05, means ± SEM, *n* = 6).

The alpha diversity of microbiota is displayed in Figure 5. Compared to fish with C group, fish from the FK3, FK6, and FK9 groups showed higher microbial richness, estimated as observed richness and Chao 1 index (Figure 5A,B, *p* < 0.05). Further, FK-fed groups tended to have higher microbial diversity (Shannon and Simpson index) compared to the C group, but no statistically significant differences were found between dietary treatments (Figure 5C,D, *p* > 0.05).

**Figure 6:**
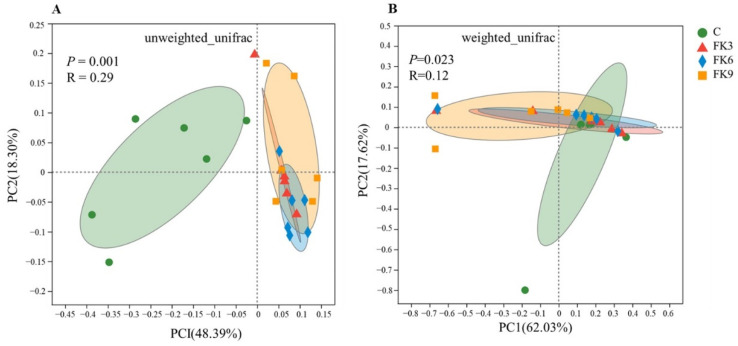
Beta diversity of microbiota in distal intestinal of largemouth bass fed diets with various FK levels. (**A**) PCoA plots based on Unweighted UniFrac show the clustering between treatments. (**B**) PCoA plots based Weighted UniFrac show the clustering between treatments.

Results from the permutation multivariate analysis of variance (PERMANOVA) analysis of weighted and unweighted UniFrac showed significant differences in gut micro-biota between dietary treatments (*p* < 0.05, Figure 6). Accordingly, the principal coordi-nates analysis (PCoA) plots based on Unweighted UniFrac showed that fish fed FK groups tended to cluster together and seemed to be separated from those fed the C group (Figure 6A), while all samples tended to cluster together based on the PCoA plots of Weighted UniFrac (Figure 6B).

**Figure 7:**
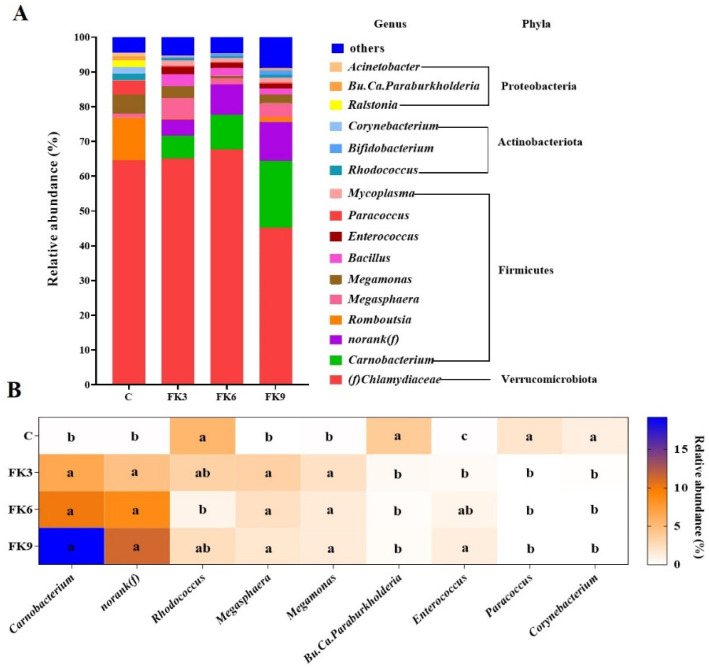
Distal intestinal microbiota composition of largemouth bass fed diets with various FK levels. (**A**) The top 16 most abundant taxa at genus level among dietary treatments. The top 16 genera were selected accounting for more than 90% of the total abundance in each treatment. (**B**) The heatmap showing significant difference in the relative abundance of 9 genera between dietary treatments (*p* < 0.05, means ± SEM, *n* = 6).

Overall, regardless of dietary treatments, the gut microbiota of largemouth bass was strongly dominantly by phylum Verrucomicrobiota followed by phyla Firmicutes, Actinobacteriota and Proteobacteria, together accounting for more than 90% of the total relative abundance (Figure 7A). The most abundant family within the phylum Verrucomicrobiota was the *Chlamydiaceae* that accounted for about 50% of the total abundance among dietary treatments, but no significant difference in its abundance between dietary treatments was observed. Significant differences in relative abundance of 8 genera and 1 family bacteria among dietary treatments were noted (Figure 7B). Specifically, higher relative abundance of genera *Carnobacterium*, *Megasphaera* and *Megamonas*, as well as family *norank(f)*, while lower relative abundance of genera *Rhodococcus*, *Bu.Ca.Paraburkholderia*, *Paracoccus* and *Corynebacterium* were observed in the FK-fed diets compare to the C group. Fish showed an increased relative abundance of genera Enterococcus with dietary the increasing levels of FK (Figure 7B).

**Figure 8:**
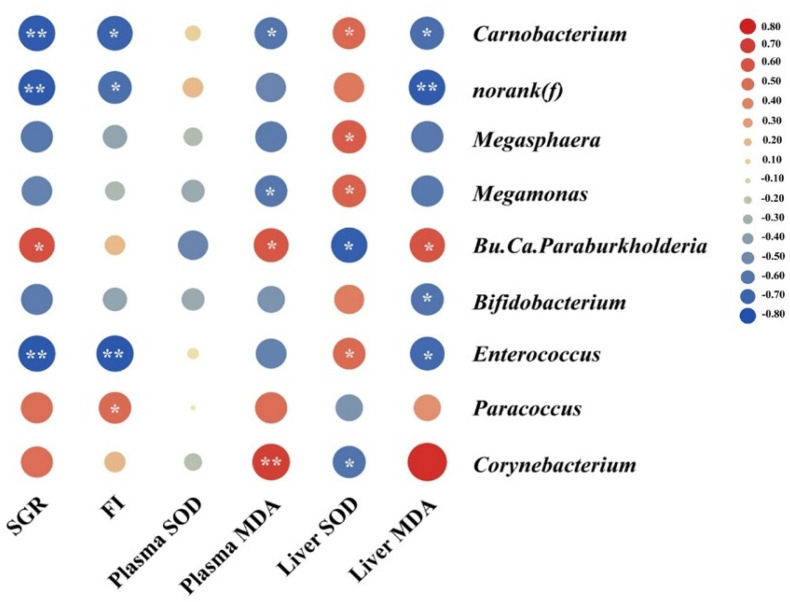
The Pearson correlation between gut microbiota and biomarkers. Circles in the balloon plot denoting the direction of the association are colored red (positive) or blue (negative) and overlaid with the symbol “*” indicating a significant correlation. Values marked with white symbol “*” are significantly correlation (*p* < 0.05), and “**” are very significantly correlation (*p* < 0.01).

For the results of Pearson correlation analysis, SGR and FI showed a strong negative relationship with the relative abundance of *Carnobacterium*, *Enterococcus* and *norank(f)* (family) (*p* < 0.01, Figure 8). The plasma and hepatic MDA levels showed positive correlations with the relative abundance of *Corynebacterium*, *Bu.Ca.Paraburkholderia* but negatively related with *Carnobacterium* (*p* < 0.01, Figure 8). Further, the hepatic MDA level showed negative correlations with the relative abundance of *norank(f)* (family), *Bifidobacterium* and *Enterococcus*. Additionally, the liver SOD levels showed a clear positive correlation with the relative abundance of *Carnobacterium*, *Megasphaera*, *Megamonas* and *Enterococcus*, but negatively correlated with *Bu.Ca.Paraburkholderia* and *Corynebacterium*.

## 4. Discussion

Over the last decade, the farming of largemouth bass has progressed from feeding animals with trash fish to feeding them with artificial diets containing high levels of FM and protein, but low starch [42,43]. As well as the inclusion of protein from traditional sources such as soy or territorial animal protein, various novel protein sources, such as *Clostridium autoethanogenum* protein [31,44,45], cottonseed protein concentrate [32], and *Methylococcus capsulatus* (FeedKind^®^) [19], have been successful in partial replacing FM in diets of largemouth bass. Although several studies have shown that FM inclusion can be reduced to 15–20% in the formulation without negative effect on growth performance [31,32], the amount of FM in largemouth bass commercial diets is generally kept at 30–50%. The previous study showed 12.9% FK was able to replace 15% FM in diet of largemouth bass fry (IBW of 5.07–5.12 g and FBW of 29.04–34.6 g), and the lowest FM inclusion level that did not affect fish health or growth was 31% [19]. In the present study, we further reduced the FM level and gradient replaced by 3%, 6% and 9% of FK. Despite the inclusion of less than 30% FM in the FK3 (29% FM) and in the FK6 (26% FM), there were no significant differences in WGR growth parameters in either the C, FK3 or FK6 groups, suggesting that FM can be successfully replaced with FK at these higher levels. However, replacement of FM with 9% FK did not result in favorable growth performance, suggesting an optimal substitution percentage is likely between these inclusion levels. In addition, although all groups showed a greater than 85% survival rate, the survival rate of the FK3, FK6, and FK9 was significantly higher than that of the C (*p* < 0.05). It should be noted that there is precedent for SCP sources, and in particular *M. capsulatus*, to increase survival [22] and to reduce gastroenteritis [25,46,47,48].

The digestibility of the feed is one of the important components in development of feeds as it affects the amount of nutrients absorbed by animals per quantity of feed ingested. The higher the digestibility for any dietary component, the more efficient the diet can be considered. The results of the current study indicate that the apparent digestibility of gross energy, protein, as well asl dry matter of the FM replacement groups were significantly higher than the C group. In contrast, it has been reported that the FM protein was replaced up to 30% by bacterial protein meal in the diet without significant effect on the digestibility in Japanese yellowtail [17]. Similarly, in seawater reared Atlantic salmon, there was no impact on apparent digestibility when comparing diets contained 0, 10 or 20% bacterial protein meal [49]. In contrast, there was a decrease in digestibility with increasing dietary bacterial protein meal in freshwater reared salmon fed experimental diets containing bacterial protein meal [13]; specifically, nitrogen digestibility decreased from 89.9% in diets containing 0% bacterial protein meal, compared to 88.1, 88.3, 86.7 and 84.2% in diets containing 6.25, 12.5, 25 and 50% bacterial meal. Furthermore, there was a significant reduction in the apparent digestibility coefficients of nitrogen, lipid, energy and amino acids when the dietary bacterial protein meal was included at 27% of the diet compared to the control diet [50]. However, some studies have shown that, due to the removal of most of the anti-nutritional factors, soluble carbohydrates, and crude fiber, SPC does not have a significant impact on the apparent digestibility of crude protein [51,52]. Based on the current study, it is suggested that FK can improve the digestive ability of largemouth bass.

Palatability could be the main reason for the low growth performance in fish fed with alternative diets [8,53]. Generally, the suppressed growth performance of carnivorous fish fed plant protein-based diets is because of the reduced feed intake as a result of palatability issues [54]. However, based on feed intake data, earlier work has shown conflicting information regarding the palatability of bacterial protein meal. For example, there is no effect on feed intake of Atlantic salmon and Atlantic halibut when 9% of the FM was replaced by bacterial protein meal [49,55,56,57]. However, when *M. capsulatus* levels were increased to 18% in Atlantic halibut (*Hippoglossus hippoglossus*) diets, feed intake and growth was reduced in fish compared to the control group [57]. In the current study, although largemouth bass ingested diets containing 9% FK, feed intake and growth performance were significantly reduced, suggesting that the largemouth bass is more sensitive to FK at relatively high inclusion levels. One possible explanation for the increased inappetence may be due to the increasing expression of α-melanocyte-stimulating hormones (α-MSH) which regulates appetite by inhibiting feeding via its binding to the melanocortin 4 receptor (MC4R), with agouti-related peptide (AgRP) that acts as an endogenous antagonist of α-MSH at MC4R [58,59]. Intracerebroventricular injections of a melanocortin receptor agonist (MTII) decrease feed intake of juvenile rainbow trout, whereas injections antagonists (HS024 and SHU9119) could increase feed intake [60]. Although not tested here, it is possible that inclusion of palatability enhancers or taste masking components in the diet may help overcome the reduced palatability at higher FK inclusion levels.

Compared to fish meal, FK contains high levels of nucleic acids and copper, which may elicit negative responses on feed intake and utilization of the diets. High dietary levels of brewer yeast (≥500 g/kg), which contained 2.5–10% ribose nucleic acid, could result in reduced palatability and growth in rainbow trout [61]. Since copper is an element required for the bacterial methane oxidase in the bacterial protein meal production process [62], compared to other feed ingredients, the copper content of FK is relatively high, about 101 mg/kg (Calysta data). Once FK is incorporated into the feed, copper levels are markedly lower at 7.9–16.4 mg per kg of feed in the current study. Whilst feed intake rates were significantly reduced in Indian major carp (*Cirrhinus mrigala* Hamilton) when exposed to 0.02, 0.10, 0.15 and 0.23 mg L^−1^ waterborne copper [63], no apparent negative effects, including copper accumulation, were noted in largemouth bass or common carp (*Cyprinus carpio*) exposed to high copper (290 μg·g^−1^ dry weight) in freshwater reservoirs [64]. Another negative effect of high levels of diet-borne Cu is lipid peroxidation in the internal organs [65]. Although Cu is an essential micro-nutrient for fish, Cu requirements differ among species and even within different life stages of a single species e.g., salmon—10 mg/kg feed, channel catfish—5 mg/kg feed [65]. The significant increase of apparent digestibility coefficient of Cu indicated that the high concentration of dietary Cu is readily excreted and do not accumulate in the body of largemouth bass. However, we suspect that the gradient increases of ash in body composition the fish was due to the increase of Cu deposition in the bones. Copper sources of inorganic (CuSO_4_) and organic (copper-ethanolamine) showed no significant differences in the growth performance in red drum (*Sciaenops ocellatus*) with no detrimental effects being observed in red drum fed diets supplemented with 20 mg Cu/kg, which mainly excreted via the feces [66]. Highest tissue copper concentrations were observed in fish fed diets supplemented with 10 mg Cu/kg of dry diet compared to diets containing 5 or 20 mg/kg Cu [66].

A positive effect of Cu is to improve the antioxidant capacity of the largemouth bass. Dietary Cu could enhance antioxidant defense in the hepatopancreas and intestine, improve growth, digestive and absorptive capacity, as well as decrease lipid peroxidation and protein oxidation in grass Carp (*Ctenopharyngodon idella*) [67]. SOD is a pivotal enzyme reflecting the ability to scavenge the oxygen-free radicals [68] whilst MDA is the end product of polyunsaturated fatty acid [69]. Compared to the C group, the content of MDA in the FK3, FK6 and FK9 groups decreased significantly, whilst the activity of SOD increased significantly. The content of ROS in plasma is reduced compared to the C group. It is suggested that the antioxidant activity of largemouth bass could be significantly enhanced by FK. Keap1 is an important regulator in response to cellular oxidative stress, which binds to Nrf2 and promotes its degradation in cytoplasm and translocate into the nucleus to promote downstream antioxidant genes transcription [70]. Plant protein diet suppressed immune function by inhibiting anti-oxidation, apoptosis and immunity responses in amur sturgeon (*Acipenser schrenckii*), with a significant downregulation of *Keap1* [71]. HIF1α, a key transcriptional regulator of the adaptive response to hypoxia, can also be activated by oxygen-independent regulatory pathways such as growth factors, immune cytokines and antioxidant factors [72]. The slc38a9 mutation inhibited the HIF1α protein level, which furtherly caused hypoxic stress dysregulation and reduced the ability to tolerate hypoxia in zebrafish (*Danio rerio*) [73]. Keap1-NRF2 activation is associated with HIF1α upregulation in selected cancer types [72]. In this study, compared to the largemouth bass in the C group, the intestinal antioxidant capacity of those in the FK3 group was enhanced with a significant upregulation of *Keap1* at mRNA levels and a significant downregulation of the HIF1α protein expression in distal intestine tissue.

Liver function and metabolites in plasma are important tools for the indication of the health condition of fish [74,75]. The activities of plasma ALT and AST are proposed as the indicators of liver damage and chronic hepatitis [76], and plasma TBA and AKP are clinical biomarkers of cholestasis [77,78]. This study showed that an appropriate amount of FK can reduce the activity of ALT in the serum of largemouth bass without changing the activity of AKP, which showed that adding an appropriate amount of FK has positive effects on liver health. As one of the commercial bacterial protein meals, FK is mainly produced by the continuous fermentation in natural gas and ammonia by *Methylococcus capsulatus*. *Methylococcus capsulatus* is a Gram-negative bacterial, and bacterial LPS is the main component of the outer membrane of Gram-negative bacteria and represents a “double-edged sword”, which can act as bacterial endotoxin to release endogenous active factors [79], also known as microbial molecular signals responsible for activation of the innate immune system [80]. The results of this study showed that the content of LPS in the feed and the content in plasma increased with the increase of FK in the feed. IgM is the most widely studied immunoglobulin in teleost [81]. The increased IgM, complement C3 content in plasma of FK6 and FK9 groups may be related to the increased lipopolysaccharide (LPS) content in FK. It is suggested that the LPS could activate immune responses with a corresponding increase in energy requirement in the host. Diversion of energy into mounting an immune response even at a low level, may reduce the amount of nutrients deposited within the animal. However, the reduced feed intake in the FK9 group is likely to be the most important explanatory factor for the lower growth performance in that group.

Regarding gut microbiota, the substantially higher microbial richness was observed in fish fed diets with FK compared to the C group. Microbial diversity showed a similar picture, although this was not statistically significant. Our findings suggest that dietary FK may imply a more functional gut, since a high microbial diversity is commonly taken as an indicator of normal and healthy physiological intestinal functions, whereas low diverse microbiota is generally connected with dysbiosis [82]. A similar effect was noted in Pacific white shrimp where the inclusion of 10.5% FK replacing 45% FM in the diet led to an increased microbial diversity and healthier animals [21]. These authors suggested that a higher microbial diversity could be related to high level of nucleotides in FK as nucleotides are more readily available for microbiota [21]. Notably, our previous studies suggest that the characterization of the correlation between gut microbiota and host physiological functions is an important and necessary step to identify the potential beneficial bacteria strains that could benefit host health and welfare [83,84,85]. In the present study, one of major genera, the gram-positive rod-shaped lactic acid bacteria *Carnobacterium*, was negatively correlated with growth rate, feed intake, as well as the levels of MDA in plasma and liver. The negative correlation with growth and higher abundance of *Carnobacterium* was noted to the FK9 group, which showed unfavorable growth rate compared to the other groups. Regarding the negative relation between *Carnobacterium* and the levels of plasma and liver MDA, this suggests that dietary FK might increase *Carnobacterium* abundance, and then regulate antioxidant capacity. Additionally, the relative abundance of *Bifidobacteria* was negatively related to the levels of liver MDA. The *Bifidobacteria* is one of best-known probiotic strains, has been used because of its antioxidant properties and because its role in preventing oxidative stress has been reported [86,87]. Our recent study also showed that liver MDA levels was significantly correlated with the relative abundance of bacteria, including *Pseudomonas* and *Psychrobacter* [85]. Unfortunately, the potential mechanisms behind these correlations remain unknown, but it is assumed that these could be attributed to the high level of nucleotides [14,88,89] and other potential functional components, such as large molecules and water insoluble components [24], which may regulate antioxidant relevant bacteria, and then improve the oxidative stress status. Nevertheless, these findings require further investigation to improve the knowledge on the relationship between antioxidant capacities and gut microbiota.

## 5. Conclusions

In conclusion, the results indicate that high FM diet (C with 42% FM, 15% SPC) is not best choice for largemouth bass compared to the FK3 (29% FM, 25% SPC and 3% FK) and FK6 (26% FM, 25% SPC and 6% FK). Although inclusion of 9% FK in the diet significantly reduced feed intake and thus growth performance the use of attractants to negate the effect of high FK inclusions on feed intake allowed an increase in the amount of FK that could be included. This would, however, need to be balanced with the potential negative effects of increased copper and LPS in the diet. Replacing fishmeal with 3–6% FeedKind^®^ in the diet of largemouth bass improves growth, nutrients digestibility and liver functions via the regulation of antioxidant capacity and microbiota and efforts should be made to further refine inclusion rates and to test diets under field conditions. Moreover, FeedKind^®^ protein is produced with both biogas and renewable electricity and has minimal land use and water requirements compared to terrestrial based feed ingredients, which has a carbon footprint that is comparable to or better than many other feed sources. Hence, the development of nutrient-balanced and economical sustainability FeedKind^®^ provided huge potential as fishmeal alternatives in aquaculture.

## Figures and Tables

**Table 1:** Formulation and composition of experimental diets (g/kg).

Ingredients	C	FK3	FK6	FK9
Fishmeal ^a^	420	290	260	230
FeedKind^®^^b^	0	30	60	90
Soy protein concentrated ^c^	150	250	250	250
Soybean meal	60	60	60	60
Wheat gluten meal	30	30	30	30
Spay-dried blood cell powder	50	50	50	50
Wheat flour	70	70	70	70
Cassava meal	50	50	50	50
Microcrystalline cellulose	54.3	33.7	31.7	29.6
Kelp meal	15	15	15	15
Ca(H_2_PO_4_)_2_	5.5	12.3	13	13.7
Lecithin oil	20	20	20	20
Fish oil	20.2	31.2	32.2	33.2
Soybean oil	40	40	40	40
Vitamin and mineral premix ^d^	14	14	14	14
*L*-Lysine	0	1.8	2.1	2.5
*DL*-Methionine	0	1	1	1
Y_2_O_3_	1	1	1	1
Total	1000	1000	1000	1000
Analyzed chemical compositions (g/kg, in as is basis)				
Crude protein	502	500	498	500
Crude lipid	112	113	111	120
Moisture	34.4	33.8	42.7	49.7
Crude ash	110	103	97.8	95.6
Copper (Cu, mg/kg)	3.88	7.90	11.6	16.4
Gross energy/(MJ/kg)	20.6	20.3	20.8	19.9

^a^ Fish meal: steam dried, prime. Tecnológica de Alimentos S.A., Ltd. (San Borja, Lima, Peru). ^b^ Feedkind^®^: a bacterial meal consisted of inactivated and spray-dried biomass of the aerobic bacteria *Methylococcus capsulatus* (Bath), *Cupriavidus* sp., *Aneurinibacillus danicus* and *Brevibacillus agri*. Calysta, Inc. (San Mateo, CA, USA). ^c^ Cottonseed protein concentrate was purchased from Xinjiang Jinlan plant protein Co., Ltd. (Shihezi, Xinjiang, China). ^d^ Vitamin and mineral premix (mg/kg diet): Vitamin A 20; Vitamin B1 12; Vitamin B2 10; Vitamin B6 15; Vitamin B12 (1%) 8; Niacinamide 100; Ascorbic acid (35%) 1000; Calcium pantothenate 40; Biotin (2%) 2; Folic acid 10; Vitamin E (50%) 400; Vitamin K3 20; Vitamin D3 10; Inositol 200; Choline chloride (50%) 4000; Corn gluten meal 150; FeSO_4_·H_2_O 300; ZnSO_4_·H_2_O 200; MnSO_4_·H_2_O 100; KI (10%) 80; Na_2_SeO_3_ (10% Se) 67; CoCl_2_·6H_2_O (10% Co) 5; NaCl 100; Zeolite 638.

**Table 2:** Amino acid composition of experimental diets (g/kg diet, in as is basis).

	C	FK3	FK6	FK9
Essential amino acid				
Arginine	30.3	31.8	32.1	32.1
Histidine	17.2	17.1	16.8	16.6
Isoleucine	19.8	20.9	21.1	20.8
Leucine	41.9	42.3	42.7	42.3
Lysine	36.2	36.1	36.2	35.9
Methionine	11.2	11.2	11.3	11.3
Phenylalanine	24.1	25.3	25.5	25.1
Threonine	21.0	20.5	20.3	20.5
Valine	26.4	27.5	27.7	27.5
Non-essential amino acid				
Alanine	29.3	28.6	28.8	28.8
Aspartic acid	50.8	52.0	52.4	51.8
Cysteine	6.0	5.9	6.2	5.8
Glycine	26.8	26.1	25.8	25.6
Glutamic acid	79.4	81.6	81.9	80.1
Proline	24.2	25.5	24.6	24.6
Serine	22.9	22.8	23.0	26.5

**Table 3:** Primer sequences for RT-qPCR.

Genes	Forward Primer (5′-3′)	Reverse Primer (5′-3′)	Tm (°C)	E-Values (%)	Accession Number
*EF1α*	TGCTGCTGGTGTTGGTGAGTT	TTCTGGCTGTAAGGGGGCTC	60.4	102.8	119901934
*Keap1*	TCATTGGGGAATCACATCTTTG	TGTCCAGAAAAGTGTTGCCATC	56.5	99.6	119899192
*Nrf2*	CAGACGGGGAAACAAACAATG	GGGGTAAAATACGCCACAATAAC	55.0	96.8	119905458
*CREB*	GGAGTCTGTATCGCTCAGCC	ACCAACGTAACTGTGGGACC	60.0	97.0	119904409

*EF1α*, elongation factor-1α; *Keap1*, kelch-like ECH associated protein 1; *Nrf2*, nuclear factor-erythroid factor 2; *CREB*, cAMP responsive element binding protein.

**Table 4:** Growth performances and morphometric parameters of largemouth bass fed diets with various FK levels (means ± SEM, *n* = 4).

	C	FK3	FK6	FK9
Growth performance				
SR (%) ^1^	85.00 ± 2.52 ^b^	94.00 ± 2.00 ^a^	96.00 ± 1.63 ^a^	96.00 ± 0.00 ^a^
WGR (%) ^2^	199.1 ± 10.7 ^a^	202.9 ± 7.70 ^a^	214.2 ± 9.79 ^a^	158.2 ± 8.06 ^b^
FCR ^3^	1.22 ± 0.07	1.16 ± 0.04	1.13 ± 0.02	1.22 ± 0.07
PPV (%) ^4^	27.3 ± 1.4	28.7 ± 3.2	28.8 ± 4.7	27.0 ± 5.7
PLV (%) ^5^	78.0 ± 3.7 ^ab^	82.0 ± 1.8 ^b^	93.3 ± 4.1 ^c^	69.3 ± 3.1 ^a^
Morphometric parameters				
CF (g/cm^3^) ^6^	1.94 ± 0.03 ^a^	1.83 ± 0.03 ^b^	1.86 ± 0.03 ^ab^	1.76 ± 0.02 ^b^
VSI (%) ^7^	7.03 ± 0.18	7.06 ± 0.23	7.67 ± 0.30	7.49 ± 0.19
HSI (%) ^8^	1.49 ± 0.05	1.50 ± 0.08	1.48 ± 0.07	1.50 ± 0.06
VAI (%) ^9^	2.72 ± 0.20	2.42 ± 0.14	2.67 ± 0.23	2.47 ± 0.15

Superscript lowercase letters “a, b, c” indicate significant differences (*p* < 0.05) among groups. ^1^ SR (survival rate, %) = 100 × final fish number/initial fish number. ^2^ WGR (weight gain rate, %) = 100 × [(W_f_ − W_i_)/W_i_]. ^3^ FCR (feed conversion ratio) = feed consumption/(W_f_ − W_i_). ^4^ PPV (productive protein value, %) = 100 × (FBW × C_fp_ − IBW × C_ip_)/feed protein consumption. ^5^ PLV (productive lipid value, %) = 100 × (FBW × C_fl_ − IBW × C_il_)/feed lipid consumption. ^6^ CF (condition factor) = 100 × (body weight, g)/(body length, cm)^3^. ^7^ VSI (viscera somatic index, %) = 100 × viscera weight/whole body weight. ^8^ HSI (hepatosomatic index, %) = 100 × liver weight/whole body weight. ^9^ VAI (visceral adipose index, %) = 100 × visceral adipose weight/whole body weight. W_f_: final total weight, Wi: initial total weight; C_fp_ (%) is final protein content in whole body of fish, C_ip_ (%) is initial protein content in whole body of fish; C_fl_ (%) is final lipid content in whole body of fish, C_il_ (%) is initial lipid content in whole body of fish.

**Table 5:** Macro-nutrient compositions and Cu content in whole body, liver tissue and feces of largemouth bass fed diets with various FK levels (in wet basis, means ± SEM. *n* = 4).

	C	FK3	FK6	FK9
Whole body				
Moisture (%)	68.4 ± 0.1	68.4 ± 0.3	68.3 ± 0.30	68.7 ± 0.3
Ash (%)	3.75 ± 0.03 ^c^	3.85 ± 0.07 ^bc^	3.97 ± 0.04 ^ab^	4.06 ± 0.03 ^a^
Crude protein (%)	16.9 ± 0.1	16.9 ± 0.1	16.7 ± 0.2	17.0 ± 0.0
Crude lipid (%)	9.59 ± 0.09	9.72 ± 0.30	9.77 ± 0.23	9.21 ± 0.18
Cu (mg/kg)	0.43 ± 0.00	0.50 ± 0.02	0.46 ± 0.04	0.42 ± 0.01
Liver				
Crude lipid (%)	3.69 ± 0.54 ^a^	2.84 ± 0.16 ^b^	2.99 ± 0.18 ^b^	2.84 ± 0.14 ^b^
Cu (mg/kg)	13.6 ± 1.20	14.2 ± 1.17	12.4 ± 0.88	9.55 ± 2.23
Feces				
Cu (mg/kg)	12.2 ± 1.41 ^c^	16.6 ± 0.43 ^b^	17.2 ± 1.26 ^b^	25.0 ± 0.59 ^a^

Superscript lowercase letters “a, b, c” indicate significant differences (*p* < 0.05) among groups.

**Table 6:** Apparent digestibility coefficient of nutrients in largemouth bass fed diets with various FK levels (%, means ± SEM. *n* = 4).

	C	FK3	FK6	FK9
ADC_d_ ^1^	75.9 ± 0.3 ^b^	78.0 ± 0.3 ^a^	77.8 ± 0.3 ^a^	77.8 ± 0.3 ^a^
ADC_p_ ^2^	91.2 ± 0.1 ^b^	92.2 ± 0.1 ^a^	92.0 ± 0.2 ^a^	91.9 ± 0.1 ^a^
ADC_e_ ^2^	82.3 ± 0.2 ^b^	83.7 ± 0.2 ^a^	83.6 ± 0.2 ^a^	82.7 ± 0.1 ^b^
ADC_c_ ^2^	23.2 ± 9.93 ^b^	53.3 ± 0.60 ^a^	66.5 ± 2.46 ^a^	65.8 ± 0.58 ^a^

Superscript lowercase letters “a” or “b” indicate significant differences (*p* < 0.05) among groups. ^1^ ADC_d_: apparent digestibility coefficient of dry matter = 100 × [1 − marker (Y_2_O_3_) in diet/marker (Y_2_O_3_) in feces]. ^2^ ADC of nutrients (ADC_p_, ADC_e_, and ADC_c_): apparent digestibility coefficient of nutrients (protein, energy or Cu) = 100 × [1 − (nutrients in feces/nutrients in diets) × (marker (Y_2_O_3_) in diet/marker (Y_2_O_3_) in feces)].

**Table 7:** Liver function and metabolites in plasma of largemouth bass fed diets with various FK levels (means ± SEM, *n* = 12).

	C	FK3	FK6	FK9
Liver function				
ALT (U/L)	16.4 ± 1.9 ^a^	8.41 ± 1.6 ^b^	9.14 ± 1.5 ^b^	12.2 ± 2.5 ^ab^
AST (U/L)	8.24 ± 0.78	6.47 ± 0.75	8.53 ± 0.63	8.33 ± 1.07
AKP (U/L)	84.2 ± 10.1	66.9 ± 8.8	84.0 ± 11.3	52.7 ± 7.7
TBA (μmol/L)	3.16 ± 0.85 ^ab^	3.52 ± 0.74 ^ab^	2.42 ± 0.31 ^b^	4.53 ± 0.41 ^a^
Metabolites				
Glucose (mmol/L)	4.75 ± 0.65 ^b^	5.51 ± 0.76 ^ab^	7.16 ± 0.75 ^a^	6.83 ± 0.78 ^ab^
TP (g/L)	23.2 ± 1.0 ^a^	23.7 ± 0.7 ^a^	20.6 ± 0.7 ^b^	21.5 ± 0.7 ^ab^
TC (mmol/L)	8.07 ± 1.22	7.67 ± 1.23	7.54 ± 1.14	7.40 ± 1.12
Triglyceride (mmol/L)	3.09 ± 0.53	2.48 ± 0.49	4.01 ± 0.64	2.83 ± 0.34
HDL-C (mmol/L)	2.61 ± 0.15	2.21 ± 0.18	2.21 ± 0.09	2.30 ± 0.19
HDL-C/TC	0.37 ± 0.06	0.34 ± 0.05	0.35 ± 0.06	0.35 ± 0.04
LDL-C (mmol/L)	0.45 ± 0.03	0.37 ± 0.05	0.38 ± 0.04	0.32 ± 0.03
LDL-C/TC	0.06 ± 0.01	0.05 ± 0.01	0.06 ± 0.01	0.05 ± 0.01
NEFA (mEq/L)	0.40 ± 0.04	0.31 ± 0.05	0.27 ± 0.04	0.28 ± 0.05

Superscript lowercase letters “a” or “b” indicate significant differences (*p* < 0.05) among groups. ALT, alanine aminotransferase; AST, aspartate aminotransferase; TBA, total bile acid; AKP, alkaline phosphatase; TP, total protein; TC, total cholesterol; HDL-C, high density lipoprotein cholesterol; LDL-C, low-density lipoprotein cholesterol; NEFA, nonestesterified fatty acid.

**Table 8:** Antioxidant response in plasma and hepatic tissue of largemouth bass fed diets with various FK levels (means ± SEM. *n* = 12).

	C	FK3	FK6	FK9
Plasma				
MDA (nmol/mL)	29.56 ± 3.10 ^a^	21.39 ± 2.59 ^b^	18.23 ± 0.99 ^b^	21.77 ± 1.66 ^b^
SOD (U/mL)	8.14 ± 2.44	9.27 ± 1.55	10.73 ± 1.46	11.84 ± 1.95
SOD/MDA	0.30 ± 0.11 ^c^	0.43 ± 0.06 ^b^	0.58 ± 0.07 ^a^	0.54 ± 0.07 ^a^
Liver				
MDA (nmol/mg prot)	14.8 ± 2.87 ^a^	5.76 ± 0.58 ^b^	2.78 ± 0.48 ^c^	2.90 ± 0.62 ^c^
SOD (U/mg prot)	13.6 ± 1.04 ^b^	18.6 ± 1.20 ^ab^	17.7 ± 2.24 ^ab^	22.8 ± 2.91 ^a^
SOD/MDA	6.42 ± 1.44 ^c^	17.3 ± 2.79 ^b^	39.6 ± 8.75 ^a^	39.3 ± 7.49 ^a^

Superscript lowercase letters “a, b, c” indicate significant differences (*p* < 0.05) among groups. ROS, reactive oxygen species. MDA, malondialdehyde; SOD, superoxide dismutase.

## Data Availability

Data is contained within the article.

## References

[1] Miles R.D., Chapman F.A. (2006). The benefits of fish meal in aquaculture diets. EDIS UF/IFAS Ext..

[2] Lu F., Haga Y., Satoh S. (2015). Effects of replacing fish meal with rendered animal protein and plant protein sources on growth response, biological indices, and amino acid availability for rainbow trout *Oncorhynchus mykiss*. Fish. Sci..

[3] Food and Agriculture Organization of the United Nations (FAO) (2020). The State of World Fisheries and Aquaculture 2020. Sustainability in Action.

[4] Goytortúa-Bores E., Civera-Cerecedo R., Rocha-Meza S., Green-Yee A. (2006). Partial replacement of red crab (*Pleuroncodes planipes*) meal for fish meal in practical diets for the white shrimp *Litopenaeus vannamei*. Effects on growth and in vivo digestibility. Aquaculture.

[5] Kaushik S.J., Seiliez I. (2010). Protein and amino acid nutrition and metabolism in fish: Current knowledge and future needs. Aquac. Res..

[6] Hardy R.W., Brezas A. (2022). Diet formulation and manufacture. Fish Nutrition.

[7] Barroso F.G., de Haro C., Sánchez-Muros M.-J., Venegas E., Martínez-Sánchez A., Pérez-Bañón C. (2014). The potential of various insect species for use as food for fish. Aquaculture.

[8] Jones S.W., Karpol A., Friedman S., Maru B.T., Tracy B. (2020). Recent advances in single cell protein use as a feed ingredient in aquaculture. Curr. Opin. Biotechnol..

[9] Øverland M., Tauson A.H., Shearer K., Skrede A. (2010). Evaluation of methane-utilising bacteria products as feed ingredients for monogastric animals. Arch. Anim. Nutr..

[10] Dalton H. (2005). The Leeuwenhoek Lecture 2000 the natural and unnatural history of methane-oxidizing bacteria. Philos. Trans. R. Soc. Lond. B Biol. Sci..

[11] Hanson R.S., Hanson T. (1996). Methanotrophic bacteria. Microbiol. Rev..

[12] Sharif M., Zafar M.H., Aqib A.I., Saeed M., Farag M.R., Alagawany M.J.A. (2021). Single cell protein: Sources, mechanism of production, nutritional value and its uses in aquaculture nutrition. Aquaculture.

[13] Storebakken T., Baeverfjord G., Skrede A., Olli J.J., Berge G.M. (2004). Bacterial protein grown on natural gas in diets for Atlantic salmon, *Salmo salar*, in freshwater. Aquaculture.

[14] Skrede A., Berge G., Storebakken T., Herstad O., Aarstad K., Sundstøl F. (1998). Digestibility of bacterial protein grown on natural gas in mink, pigs, chicken and Atlantic salmon. Anim. Feed Sci. Technol..

[15] Øverland M., Petter Kjos N., Skrede A. (2004). Effect of bacterial protein meal grown on natural gas on growth performance and carcass traits of pigs. Ital. J. Anim. Sci..

[16] Matassa S., Boon N., Pikaar I., Verstraete W. (2016). Microbial protein: Future sustainable food supply route with low environmental footprint. Microb. Biotechnol..

[17] Biswas A., Takakuwa F., Yamada S., Matsuda A., Saville R.M., LeBlanc A., Silverman J.A., Sato N., Tanaka H. (2020). Methanotroph (*Methylococcus capsulatus*, Bath) bacteria meal as an alternative protein source for Japanese yellowtail, *Seriola quinqueradiata*. Aquaculture.

[18] Xu B., Liu Y., Chen K., Wang L., Sagada G., Tegomo A.F., Yang Y., Sun Y., Zheng L., Ullah S. (2021). Evaluation of methanotroph (*Methylococcus capsulatus*, Bath) bacteria meal (FeedKind^®^) as an alternative protein source for juvenile black sea bream, *Acanthopagrus schlegelii*. Front. Mar. Sci..

[19] Zhang Q., Liang H., Longshaw M., Wang J., Ge X., Zhu J., Li S., Ren M. (2022). Effects of replacing fishmeal with methanotroph (*Methylococcus capsulatus*, Bath) bacteria meal (FeedKind^®^) on growth and intestinal health status of juvenile largemouth bass (*Micropterus salmoides*). Fish Shellfish. Immunol..

[20] Chama M.K.H., Liang H., Huang D., Ge X., Ren M., Zhang L., Wu L., Ke J. (2021). Methanotroph (*Methylococcus capsulatus*, Bath) as an alternative protein source for genetically improved farmed tilapia (GIFT: *Oreochromis niloticus*) and its effect on antioxidants and immune response. Aquac. Rep..

[21] Chen Y., Chi S., Zhang S., Dong X., Yang Q., Liu H., Zhang W., Deng J., Tan B., Xie S. (2021). Replacement of fish meal with methanotroph (*Methylococcus capsulatus*, Bath) bacteria meal in the diets of Pacific white shrimp (*Litopenaeus vannamei*). Aquaculture.

[22] Jintasataporn O., Chumkam S., Triwutanon S., LeBlanc A., Sawanboonchun J. (2021). Effects of a single cell protein (*Methylococcus capsulatus*, Bath) in Pacific white shrimp (*Penaeus vannamei*) diet on growth performance, survival rate and resistance to *Vibrio parahaemolyticus*, the causative agent of acute hepatopancreatic necrosis disease. Front. Mar. Sci..

[23] Romarheim O.H., Hetland D.L., Skrede A., Øverland M., Mydland L.T., Landsverk T. (2013). Prevention of soya-induced enteritis in Atlantic salmon (*Salmo salar*) by bacteria grown on natural gas is dose dependent and related to epithelial MHC II reactivity and CD8alpha+ intraepithelial lymphocytes. Br. J. Nutr..

[24] Romarheim O.H., Landsverk T., Mydland L.T., Skrede A., Øverland M. (2013). Cell wall fractions from *Methylococcus capsulatus* prevent soybean meal-induced enteritis in Atlantic salmon (*Salmo salar*). Aquaculture.

[25] Romarheim O.H., Øverland M., Mydland L.T., Skrede A., Landsverk T. (2011). Bacteria grown on natural gas prevent soybean meal-induced enteritis in Atlantic salmon. J. Nutr..

[26] Coyle S.D., Tidwell J.H., Webster C.D. (2000). Response of largemouth bass *Micropterus salmoides* to dietary supplementation of lysine, methionine, and highly unsaturated fatty acids. J. World Aquac. Soc..

[27] Bai J.J., Li S.J. (2019). Genetic Breeding and Molecular Marker-Assisted Selective Breeding of Largemouth Bass.

[28] Ministry of Agriculture and Rural Affairs (2020). China Fishery Statistical Yearbook.

[29] Huang D., Wu Y.B., Lin Y.Y., Chen J.M., Karrow N., Ren X., Wang Y. (2017). Dietary Protein and Lipid Requirements for Juvenile Large-mouth Bass, *Micropterus salmoides*. J. World Aquac. Soc..

[30] Cai Z.N., Qian X.Q., Xie S.Q. (2020). Optimal dietary protein concentrations for largemouth bass (*Micropterus salmoides*) of different sizes (10–500 g), *Aquacul*. Int..

[31] Ma S., Liang X., Chen P., Wang J., Gu X., Qin Y., Blecker C., Xue M. (2022). A new single-cell protein from *Clostridium autoethanogenum* as a functional protein for largemouth bass (*Micropterus salmoides)*. Anim. Nutr..

[32] Xie X., Wang J., Guan Y., Xing S., Liang X., Xue M., Wang J., Chang Y., Leclercq E. (2022). Cottonseed protein concentrate as fishmeal alternative for largemouth bass (*Micropterus salmoides*) supplemented a yeast-based paraprobiotic: Effects on growth performance, gut health and microbiome. Aquaculture.

[33] Yu H., Zhang L., Chen P., Liang X., Cao A., Han J., Wu X., Zheng Y., Qin Y., Xue M. (2019). Dietary bile acids enhance growth, and alleviate hepatic fibrosis induced by a high starch diet via AKT/FOXO1 and cAMP/AMPK/SREBP1 pathway in *Micropterus salmoides*. Front. Physiol..

[34] Liang X.F., Chen P., Wu X.L., Xing S.J., Morais S., He M.L., Gu X., Xue M. (2022). Effects of High starch and supplementation of an olive extraction the growth performance, hepatic antioxidant capacity and lipid metabolism of largemouth bass (*Micropterus salmoides*). Antioxidants.

[35] Wang J., Yun B., Xue M., Wu X., Zheng Y., Li P. (2012). Apparent digestibility coefficients of several protein sources, and replacement of fishmeal by porcine meal in diets of Japanese seabass, *Lateolabrax japonicus*, are affected by dietary protein levels. Aquac. Res..

[36] AOAC (1997). Official Methods of Analysis.

[37] Zhang Y., Chen P., Liang X.F., Han J., Wu X.F., Yang Y.H., Xue M. (2019). Metabolic disorder induces fatty liver in Japanese seabass, *Lateolabrax japonicas* fed a full plant protein diet and regulated by cAMP-JNK/NF-kB caspase signal pathway. Fish Shellfish Immun..

[38] Magoč T., Salzberg S.L. (2011). FLASH: Fast length adjustment of short reads to improve genome assemblies. Bioinformatics.

[39] Goebel S.E. (1994). A place for DNA-DNA reassorciation and 16S rRNA sequence analysis in the present species definition in bacteriology. Int. J. Syst. Bacteriol..

[40] Edgar R.C. (2013). UPARSE: Highly accurate OTU sequences from microbial amplicon reads. Nat. Methods.

[41] Wang Q., Garrity G.M., Tiedje J.M., Cole J.R. (2007). Naive Bayesian classifier for rapid assignment of rRNA sequences into the new bacterial taxonomy. Appl. Environ..

[42] Ma H.J., Mou M.M., Pu D.C., Lin S.M., Chen Y.J., Luo L. (2019). Effect of dietary starch level on growth, metabolism enzyme and oxidative status of juvenile largemouth bass, *Micropterus salmoides*. Aquaculture.

[43] Chen P., Zhu Y.P., Wu X.F., Gu X., Xue M., Liang X.F. (2018). Metabolic adaptation to high starch diet in largemouth bass (*Micropterus salmoides*) was associated with the restoration of metabolic functions via inflammation, bile acid synthesis and energy metabolism. Br. J. Nutr..

[44] Yang P., Li X., Song B., He M., Wu C., Leng X. The potential of *Clostridium autoethanogenum*, a new single cell protein, in substituting fish meal in the diet of largemouth bass (*Micropterus salmoides*): Growth, feed utilization and intestinal histology. Aquac. Fish..

[45] Zhu S., Gao W., Wen Z., Chi S., Shi Y., Hu W., Tan B. (2022). Partial substitution of fish meal by *Clostridium autoethanogenum* protein in the diets of juvenile largemouth bass (*Micropterus salmoides*). Aquac. Rep..

[46] Dabrowski K.J.R.N.D. (1984). The feeding of fish larvae: Present” state of the art” and perspectives. Reprod. Nutr. Dév..

[47] Banerjee S., Azad S., Vikineswary S., Selvaraj O., Mukherjee T. (2000). Phototrophic bacteria as fish feed supplement. Asian-Australas. J. Anim. Sci..

[48] Laranja J.L.Q., Ludevese-Pascual G.L., Amar E.C., Sorgeloos P., Bossier P., De Schryver P. (2014). Poly-β-hydroxybutyrate (PHB) accumulating *Bacillus* spp. improve the survival, growth and robustness of *Penaeus monodon* (Fabricius, 1798) postlarvae. Vet. Microbiol..

[49] Berge G.M., Baeverfjord G., Skrede A., Storebakken T. (2005). Bacterial protein grown on natural gas as protein source in diets for Atlantic salmon, *Salmo salar*, in saltwater. Aquaculture.

[50] Aas T.S., Hatlen B., Grisdale-Helland B., Terjesen B.F., Bakke-McKellep A.M., Helland S.J. (2006). Effects of diets containing a bacterial protein meal on growth and feed utilisation in rainbow trout (*Oncorhynchus mykiss*). Aquaculture.

[51] Berge G.M., Grisdale-Helland B. (1999). Soy protein concentrate in diets for Atlantic halibut (*Hippoglossus hippoglossus*). Aquaculture.

[52] Storebakken T., Shearer K.D., Roem A.J. (1998). Availability of protein, phosphorus and other elements in fish meal, soy-protein concentrate and phytase-treated soy-protein concentrate-based diets to Atlantic salmon, *Salmo salar*. Aquaculture.

[53] Liang X.F., Han J., Xue M., Yu H.H., Huang H.Y., Wu X.F., Zheng Y.H., Qin Y.C., Liang X.F. (2019). Growth and feed intake regulation responses to anorexia, adaptation and fasting in Japanese seabss, *Lateolabrax japonicas* when fishmeal is totally replaced by plant protein. Aquaculture.

[54] Nagel F., von Danwitz A., Tusche K., Kroeckel S., van Bussel C.G., Schlachter M., Adem H., Tressel R.-P., Schulz C.J. (2012). Nutritional evaluation of rapeseed protein isolate as fish meal substitute for juvenile turbot (*Psetta maxima* L.)—Impact on growth performance, body composition, nutrient digestibility and blood physiology. Aquaculture.

[55] Aas T.S., Grisdale-Helland B., Terjesen B.F., Helland S.J. (2006). Improved growth and nutrient utilisation in Atlantic salmon (*Salmo salar*) fed diets containing a bacterial protein meal. Aquaculture.

[56] Storebakken T., Kvien I., Shearer K., Grisdale-Helland B., Helland S., Berge G. (1998). The apparent digestibility of diets containing fish meal, soybean meal or bacterial meal fed to Atlantic salmon (*Salmo salar*): Evaluation of different faecal collection methods. Aquaculture.

[57] Romarheim O. (2002). Bioprotein in diets for Atlantic halibut and Atlantic salmon, with a focus on technical feed quality and growth parameters. Master’s Thesis.

[58] White S.L., Volkoff H., Devlin R.H. (2016). Regulation of feeding behavior and food intake by appetite-regulating peptides in wild-type and growth hormone-transgenic coho salmon. Horm. Behav..

[59] Metz J.R., Peters J.J., Flik G. (2006). Molecular biology and physiology of the melanocortin system in fish: A review. Gen. Comp. Endocrinol..

[60] Schjolden J., Schioth H.B., Larhammar D., Winberg S., Larson E.T. (2009). Melanocortin peptides affect the motivation to feed in rainbow trout (*Oncorhynchus mykiss)*. Gen. Comp. Endocrinol..

[61] Rumsey G.L., Kinsella J.E., Shetty K.J., Hughes S.G. (1991). Effect of high dietary concentrations of brewer’s dried yeast on growth performance and liver uricase in rainbow trout (*Oncorhynchus mykiss*). Anim. Feed Sci. Technol..

[62] Murrell J.C., McDonald I.R., Gilbert B. (2000). Regulation of expression of methane monooxygenases by copper ions. Trends Microbiol..

[63] Mohanty M., Adhikari S., Mohanty P., Sarangi N. (2009). Role of waterborne copper on survival, growth and feed intake of Indian major carp, *Cirrhinus mrigala Hamilton*. Bull. Environ. Contam. Toxicol..

[64] Anderson M.A., Giusti M.S., Taylor W.D. (2001). Hepatic copper concentrations and condition factors of largemouth bass (*Micropterus salmoides*) and common carp (*Cyprinus carpio*) from copper sulfate-treated and untreated reservoirs. Lake Reserv. Manag..

[65] Clearwater S.J., Farag A.M., Meyer J.J. (2002). Bioavailability and toxicity of dietborne copper and zinc to fish. Comp. Biochem. Physiol. Part C Toxicol. Pharmacol..

[66] Chen K., Yamamoto F.Y., Gatlin D.M. (2020). Effects of inorganic and organic dietary copper supplementation on growth performance and tissue composition for juvenile red drum (*Sciaenops ocellatus* L.). Aquac. Nut..

[67] Tang Q.Q., Feng L., Jiang W.D., Liu Y., Jiang J., Li S.H., Kuang S.Y., Tang L., Zhou X.Q. (2013). Effects of dietary copper on growth, digestive, and brush border enzyme activities and antioxidant defense of hepatopancreas and intestine for young grass carp (*Ctenopharyngodon idella*). Biol. Trace Elem. Res..

[68] Ye C.-X., Wan F., Sun Z.-Z., Cheng C.-H., Ling R.-Z., Fan L.-F., Wang A. (2016). Effect of phosphorus supplementation on cell viability, anti-oxidative capacity and comparative proteomic profiles of puffer fish (*Takifugu obscurus*) under low temperature stress. Aquaculture.

[69] Lei Y., Sun Y., Wang X., Lin Z., Bu X., Wang N., Du Z., Qin J., Chen L.J.A. (2021). Effect of dietary phosphorus on growth performance, body composition, antioxidant activities and lipid metabolism of juvenile Chinese mitten crab (*Eriocheir sinensis*). Aquaculture.

[70] Nguyen T., Nioi P., Pickett C.B. (2009). The Nrf2-antioxidant response element signaling pathway and its activation by oxidative stress. J. Biol. Chem..

[71] Wei H.C., Chen P., Liang X.F., Yu H.H., Wu X.F., Han J., Luo L., Gu X., Xue M. (2019). Plant protein diet suppressed immune function by inhibiting spiral valve intestinal mucosal barrier integrity, anti-oxidation, apoptosis, autophagy and proliferation responses in amur sturgeon (*Acipenser schrenckii*). Fish Shellfish Immunol..

[72] Lacher S.E., Levings D.C., Freeman S., Slattery M. (2018). Identification of a functional antioxidant response element at the HIF1A locus. Redox Biol..

[73] Wu X., Chen J., Liu C., Wang X., Zhou H., Mai K., He G. (2022). Slc38a9 deficiency induces apoptosis and metabolic dysregulation and leads to premature death in zebrafish. Int. J. Mol. Sci..

[74] Congleton J., Wagner T. (2006). Blood-chemistry indicators of nutritional status in juvenile salmonids. J. Fish Biol..

[75] Iwama G.K., Afonso L.O., Vijayan M.M. (1998). Stress in fish. Ann. N. Y. Acad. Sci..

[76] Nyblom H., Berggren U., Balldin J., Olsson R. (2004). High AST/ALT ratio may indicate advanced alcoholic liver disease rather than heavy drinking. Alcohol Alcohol..

[77] Wang X., Wang G., Qu J., Yuan Z., Pan R., Li K. (2020). Calcipotriol inhibits NLRP3 signal through YAP1 activation to alleviate cholestatic liver injury and fibrosis. Front. Pharmacol..

[78] Yu L.L., Yu H.H., Liang X.F., Li N., Wang X., Li F.H., Wu X.F., Zheng Y.H., Xue M., Liang X.F. (2018). Dietary butylated hydroxytoluene improves lipid metabolism, antioxidant and anti-apoptotic response of largemouth bass (*Micropterus salmoides*). Fish Shellfish Immunol..

[79] Mangoni M.L., Epand R.F., Rosenfeld Y., Peleg A., Barra D., Epand R.M., Shai Y. (2008). Lipopolysaccharide, a key molecule involved in the synergism between temporins in inhibiting bacterial growth and in endotoxin neutralization. J. Biol. Chem..

[80] Trent M.S., Stead C.M., Tran A.X., Hankins J. (2006). Invited review: Diversity of endotoxin and its impact on pathogenesis. J. Endotoxin Res..

[81] Purcell M.K., Bromage E.S., Silva J., Hansen J.D., Badil S.M., Woodson J.C., Hershberger P.K. (2012). Production and characterization of monoclonal antibodies to IgM of Pacific herring (*Clupea pallasii*). Fish Shellfish Immunol..

[82] Mosca A., Leclerc M., Hugot J.P. (2016). Gut microbiota diversity and human diseases: Should we reintroduce key predators in our ecosystem?. Front Microbiol..

[83] Wang J., Jaramillo-Torres A., Li Y., Brevik Ø.J., Jakobsen J.V., Kortner T.M., Krogdahl Å. (2022). Gut health and microbiota in out-of-season Atlantic Salmon (*Salmo salar* L.) smolts before and after seawater transfer under commercial Arctic conditions: Modulation by functional feed ingredients. Front. Mar. Sci..

[84] Wang J., Jaramillo-Torres A., Li Y., Kortner T.M., Gajardo K., Brevik O.J., Jakobsen J.V., Krogdahl Å. (2021). Microbiota in intestinal digesta of Atlantic salmon (*Salmo salar*), observed from late freshwater stage until one year in seawater, and effects of functional ingredients: A case study from a commercial sized research site in the Arctic region. Anim. Microbiome..

[85] Wang J., Mai K.S., Ai Q.H. (2022). Conventional soybean meal as fishmeal alternative in diets of Japanese Seabass (*Lateolabrax japonicus*): Effects of functional additives on growth, immunity, antioxidant capacity and disease resistance. Antioxidant.

[86] Averina O.V., Poluektova E.U., Marsova M.V., Danilenko V.N. (2021). Biomarkers and utility of the antioxidant potential of probiotic *Lactobacilli* and *Bifidobacteria* as representatives of the human gut microbiota. Biomedicines.

[87] Esteban-Torres M., Ruiz L., Lugli G.A., Ventura M., Margolles A., van Sinderen D. (2021). Editorial: Role of *Bifidobacteria* in human and animal health and biotechnological applications. Front. Microbiol..

[88] Hossain M.S., Koshio S., Kestemont P. (2019). Recent advances of nucleotide nutrition research in aquaculture: A review. Rev. Aquac..

[89] Wang J., Chen L.M., Xu J., Ma S.F., Liang X.F., Wei Z.X., Li D.M., Xue M. (2022). C1 gas protein: A potential protein substitute for advancing aquaculture sustainability. Rev. Aquac..

